# The Influence of Stigma Perceptions on Employees’ Claims Experiences for Psychological Injuries: Re-Examination of a Cross-Sectional Survey among Australian Police and Emergency Service Personnel

**DOI:** 10.3390/ijerph191912438

**Published:** 2022-09-29

**Authors:** Samineh Sanatkar, Jenn Bartlett, Samuel Harvey, Isabelle Counson, David Lawrence

**Affiliations:** 1 Black Dog Institute, School of Psychiatry, UNSW Sydney, Randwick, NSW 2031, Australia; 2 School of Population Health, Curtin University, Bentley, WA 6102, Australia

**Keywords:** first responders, workers compensation, work claims, mental health stigma, mental illness

## Abstract

While a large body of research assessed the contribution of mental health stigma on disclosure, treatment seeking, and recovery, limited research exists seeking to identify the relative contribution of stigma beliefs on workers’ compensation claims for psychological injury. Survey data of ambulance, fire and rescue, police, and state emergency service personnel (*N* = 1855, aged 45–54 years, 66.4% male) was re-examined to assesses the unique and combined associations of self-, personal, and workplace stigma with workers’ compensation claims experiences and recovery. Participants responded to self-report stigma items (predictor variables), perceived stress, fairness, and support perceptions of going through the claims process and its impact on recovery (outcome variables). Multiple regression analyses revealed that the combined stigma dimensions predicted about one fifth of the variance of claims and recovery perceptions. Organisational commitment beliefs and the self-stigma dimension of experiences with others were the two most important, albeit weak, unique predictors across outcomes. Given the small but consistent influences of organisational commitment beliefs and the self-stigma dimension of experiences with others, it seems warranted to apply workplace interventions that are looking to establish positive workplace contact and a supportive organisational culture to alleviate negative effects attributable to mental health stigma.

## 1. Introduction

Mental health stigma refers to a set of negative attitudes, stereotyped beliefs, and discriminatory behaviour directed at individuals with mental illness [1,2]. Previous research indicates that stigma adversely affects persons with mental illness. It may contribute to a lack of support, including from health professionals, experiencing social exclusion and job loss [3,4,5]. Different forms of stigma have been proposed, which relate to whether a person expresses stigmatising views themselves or assumes other people or institutions to do so [6,7]. *Personal stigma* describes how someone perceives another person who has a mental illness [7]. Personal stigma may be a result of poor knowledge about mental illness and feeling that someone with a mental health condition is perceived to be burdensome. *Workplace stigma* describes mental health stigma that occurs in the workplace specifically [7]. It assesses the degree to which an employee believes their colleagues to express mental health stigma (perceived stigma) and the degree to which an employee believes their organisation is currently taking measures (perceived organisational commitment), or should be taking measures (structural stigma), to support workplace mental health.

In addition to facing negative reactions from others, stigmatising beliefs about mental health can also be internalised as *self-stigma* [7]. Self-stigma can be expressed by an individual with mental illness in the form of shame about having mental health problems and feelings of being a burden to others or having negative experiences with others due to their mental health problems. This can have negative effects on a person’s self-esteem, self-efficacy beliefs, and is often identified as a barrier to help seeking [8,9,10]. For those who receive treatment, the presence of mental health stigma can slow recovery rates [8,11]. A longitudinal assessment of adults who were in psychiatric treatment and received disability pensions showed that participants recovered to a lesser extent after a 1-year period if they expressed self-stigma at the start of the study [11]. These results suggested that the presence of stigma may hinder recovery efforts among those who had adopted stigmatising beliefs. This raises the question as to whether mental health stigma may also negatively influence processes that workers seek adjunct to therapeutic interventions, such as issuing workplace compensation claims for psychological harm experienced at work. 

Reviews indicate that first responder groups are particularly at risk of developing mental health problems such as depression, post-traumatic stress, and alcohol use disorders [12] and are more likely than employees from other occupational sectors to file compensation claims for psychological injuries incurred at work [13]. This is because employees in police and emergency services organisations are routinely exposed to dangerous, demanding and high-stakes situations and therefore face a great number of high-intensity workplace stressors [14]. Recent research conducted in the United States found that, although the local police force regularly interacted with community members with mental illness, and were more likely to experience mental illness themselves, the prevalence of mental health stigma among the police exceeded that of the general population [15]. Police officers who had elevated levels of post-traumatic stress at the time of the investigation were particularly prone to endorse negative stereotypes about mental health [15]. This finding is contrary to earlier research findings outside the Police and Emergency Services sector suggesting that having a mental illness or being in contact with individuals with mental illness alleviates stigma [16].

The workers’ compensation process is designed to support recovery for workers who experience a work-related mental health condition. This can include allowing workers to take paid time away from their work to seek treatment and providing support in the return-to-work process. Despite being designed to actively support recovery [17], a comprehensive survey of over 14,000 Australian police and emergency service workers found that the claims process was perceived as having been a negative experience by 70% of claimants [18]. This is a considerably greater proportion compared to general Australian employees, among which only 30% of respondents reported having had neutral or negative claims experiences [19]. Related to Oexle and colleagues’ findings, police and emergency service personnel who had stigmatising views of mental illness found the claims process less helpful than those who did not endorse stigmatising views [18]. 

Based on the previous observations [11,18] and utilising the survey data collected by Lawrence, Kyron and colleagues [18,20], the current research seeks to investigate the relative contributions of different stigma types (self-stigma, personal stigma, and workplace stigma) on compensation claims processes by police and emergency service workers and the relevance of these processes on employees’ recovery. It also seeks to identify the overall contribution of mental health stigma on claims process experiences. The purpose of examining mental health stigma types simultaneously is to identify the magnitude of the combined stigma effects and to identify specific stigma dimensions that may be most important to address in targeted interventions or compensation system amendments. Further, the study looks to identify whether there are any stigma differences between employees with a mental health condition who have filed claims and those who did not. This may be useful to gauge whether claimants have different stigma perceptions due to their claims experiences. This research adopts a novel approach to examining stigma influences and is exploratory in nature. We therefore did not formulate specific hypotheses. 

## 2. Method

### 2.1. Overview

In response to evidence indicating that emergency service personnel may be at greater risk of developing a mental disorder, the Australian mental health organisation Beyond Blue established the Police and Emergency Services Program and commissioned the first large-scale national mental health survey for emergency service workers titled “Answering the Call: The National Mental Health and Wellbeing Survey of Police and Emergency Services” [21]. Survey items were developed in collaboration with subject matter experts and in line with guidelines outlined in the Australian Good Practice Framework for Mental Health and Wellbeing in First Responders [22]. The Human Research Ethics Committee at The University of Western Australia granted ethical approval for the original protocol (protocol # RA/4/1/9036) and the Human Research Ethics Committee at UNSW Sydney approved additional analysis undertaken in the present article (protocol # HC210149). A detailed breakdown of recruitment strategies, survey questionnaires, participant characteristics, and main findings of the original protocol is published elsewhere [20,21].

### 2.2. Participants and Procedures

**Figure 1:**
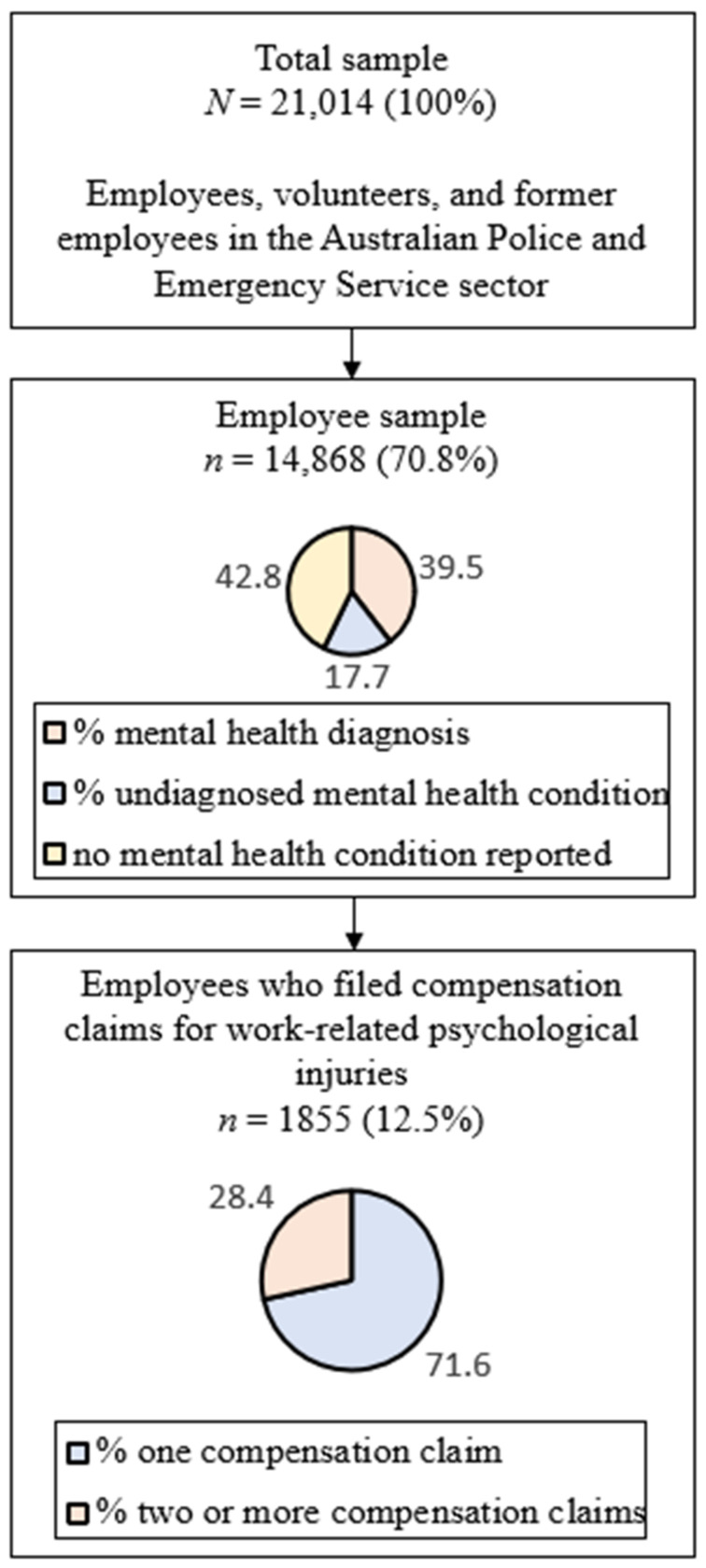
Flowchart presenting the participant composition of the *Answering the Call* survey.

Answering the Call surveyed over 20,000 employees, volunteers and former employees in Police and Emergency Services organisations across Australia. For this analysis, data from the 14,868 employees who worked at ambulance, fire and rescue, police, and state emergency services at the time of survey completion between October 2017 and March 2018 was used. A previous analysis indicated that the sample was largely representative of the Australian police and emergency services sector, with minor overrepresentations of female and older-aged employees [18]. Survey data have been weighted to represent the full population of Police and Emergency Services employees in Australia. Out of all employed respondents, 5865 (5865/14,868, 39.49%) reported having previously been diagnosed with a mental health condition by a doctor or mental health professional and a further 2633 (2633/14,868, 17.71%) reported having had an undiagnosed mental health condition in their lifetime. A subset of 1855 (1855/14,868, 12.48%) reported to have previously made one (1329/1855, 71.64%) or more (526/1855, 28.36%) compensation claims for psychological injuries obtained at work. A graphical representation of the participant composition is provided in Figure 1. 

Prior publications resulting from this data set investigated the influence of stigma on organisational help-seeking among police and emergency service workers and the prevalence of mental health stigma among those who had filed a compensation claim [18,20]. However, the analysis presented in this article differs from these previous assessments in that it will assess the unique and combined influence of stigmatising beliefs expressed within this subset of respondents with compensation claims experience for work-related psychological injuries (*N* = 1855).

### 2.3. Measures

#### 2.3.1. Compensation Claims Experiences

Respondents who indicated that they had ever made one or more compensation claims for psychological injuries at work were asked four targeted questions about their most recent claims experience. The first item queried the overall impact of the experience on respondents’ recovery (“What impact did going through the claims experience last time have on your recovery?”). Responses were on a 5-point Likert-type scale anchored 1 (*very positive impact*) and 5 (*very negative impact*). Respondents were asked to what degree they found their claims experience to be supportive (“How supportive did you find the claims experience?”) and stressful (“How stressful did you find the claims experience?”). Answers were recorded on a 5-point Likert-type scale anchored 1 (*not at all*) and 5 (*extremely*). The fourth item assessed respondents’ perceptions of fairness throughout the claims process (“How fairly do you believe you were treated when you went through the last claims experience?”) and was recorded on a 3-point response scale with response values of 1 (*not fairly at all*), 2 (*somewhat fairly*) and 3 (*very fairly*). These four items were used as single-item indicators of impact, support, perceived stress, and fairness of respondents’ most recent claims experience. Response values were thus not further coded or aggregated. 

#### 2.3.2. Stigmatising Mental Health Attitudes

Based on the types of stigmatising beliefs surrounding mental illness described by Beyond Blue [7], the Answering the Call survey delineated between self-stigma, personal stigma and workplace stigma. Employees responded to all stigma-related items on a 5-point Likert-type scale anchored 1 (*strongly disagree*) and 5 (*strongly agree*). We recoded disagreements with stigma items (response values of 1 and 2) to negative values (−1 and −2, respectively), neutral responses of 3 to 0, and agreement with items (response values of 4 and 5) to positive values (1 and 2, respectively). After recoding, scores above the neutral point (0) generally indicated the presence, or endorsement, of stigmatising beliefs, whereas scores below the neutral point indicated the absence, or rejection, of mental health stigma. To aggregate raw scores of multi-item measures, we further calculated mean scores for subscale items. 

Self-stigma items measured the dimensions of shame (e.g., “I [would] feel embarrassed about seeking professional help”), burden (e.g., “I [would] feel like a burden to other people”), and experiences with others (e.g., “I [would] feel that people avoid me because of my emotional or mental health problems). 

Personal stigma items pertaining to the ignorance (versus knowledge) dimension (e.g., “If someone is experiencing depression or anxiety, it is a sign of personal weakness”) were modified from the Depression Stigma Scale [23]. The perceived burden dimension of personal stigma was measured using items such as “I would prefer not to have someone with depression or anxiety working on the same team as me”. 

Workplace stigma items measured the dimensions of perceived stigma (e.g., “Most people in my organisation believe that people with depression or anxiety cannot be taken as seriously as other people”), structural stigma (“It is important for workplaces to support someone with a mental health condition”), and perceived organisational commitment (e.g., “I believe my organisation is committed to making changes that promote mental health and wellbeing”). Responses to the structural stigma item were reverse scored and then recoded, so that scores of 1 and 2 indicated the presence of structural stigma. Higher scores (positive mean scores) on the perceived organisational commitment dimension indicated a belief that respondents’ organisations were engaged in actions advancing mental health at the workplace. 

### 2.4. Analysis Plan

The analyses presented in this article comprised of eight independent variables and four dependent variables. Independent variables were self-stigma dimensions of shame, burden, and experiences with others, personal stigma dimensions of ignorance and perceived burden, workplace stigma dimensions of perceived stigma and structural stigma, and organisational commitment. To examine the unique and combined contributions of stigma types on employees’ experiences of the compensation process, we were looking to employ a total of four models, by which each model would contain all stigma variables and one dependent variable. Dependent variables were perceived stressfulness of the compensation claim process (Model 1), impact of the claims process on recovery perceptions (Model 2), perceived fairness (Model 3) and support (Model 4) going through the compensation claim process. 

Of the initial subset of workers with compensation claims experience (*N* = 1855), 1416 (76%) provided full data on measures of mental health stigma, claims experiences, and recovery. We conducted a power calculation to discern whether the limited sample size of 1416 of employees who had completed all stigma-related and claims experience variables was adequate to detect small effects expected in this exploratory analysis. To test this, G*Power Version 3.1.9.7 (Heinrich Heine University Düsseldorf, Germany) [24] was utilised. Results indicated that a sample size of 1145 was required to detect effects of a small magnitude with an alpha level of 0.05, a power of 0.95, and 8 predictor variables, indicating that the available sample size was appropriate. 

In preparation for further analysis steps, we examined whether stigma variables would show concerning levels of intercorrelations. To test whether multicollinearity was an issue, we first inspected intercorrelations of stigma variables via Pearson coefficients. We noted the highest correlation between the personal stigma dimensions of ignorance and perceived burden (*r* = 0.60). The correlational strength, however, remained well below the commonly used indicator of multicollinearity of 0.70 and above [25]. We then inspected collinearity statistics. Variance Inflation Factor (VIF) and Tolerance values did not indicate that the variables entered were providing redundant information. All VIF values were sufficiently low (values ranged from 1.08 to 1.88), with maximum levels remaining below conservative recommendations of under 2.5 [26]. Similarly, Tolerance values ranged from 0.53 to 0.92, exceeding recommended levels (equivalent to conservative VIF estimates) of at least 0.40 or above [26]. These results suggested that variables were not subject to collinearity concerns. We therefore proceeded with the analysis outlined below. 

We performed four multiple linear regression analyses in IBM SPSS Statistics Version 26 (IBM Corporation, New York, NY, USA) to explore the extent to which employees’ stigma beliefs influenced self-reported experiences during their claims and recovery processes for psychological injuries obtained at work. The prediction models helped discern the proportions of explained variances that could be attributed to various stigma beliefs, with the ability to distinguish between the combined and unique contributions of stigma variables. We reported the proportion of variance explained by each significant predictor relative to the total explained variance (inclusive R^2^, see also [27]). Following initial analyses containing the stigma variables, we entered potential confounds to examine whether any observed effects would be sustained. We considered respondents’ age group, gender, work sector, presence of a previous mental health diagnosis, presence of a current mental health condition, and previously having taken stress leave across all four models.

## 3. Results

### 3.1. Sample Characteristics

Employees who had made a workers’ compensation claim for psychological injuries in the past (1855/14,868, 12.5%) reported perceived stress levels above the midrange of 3 (*M* = 3.33, *SD* = 1.34) and noted an overall negative impact (values above 3) on their recovery process (*M* = 3.68, *SD* = 1.29). Perceived fairness (*M* = 1.93, *SD* = 0.75) and support (*M* = 2.10, *SD* = 1.14) through the claims process remained below the midrange, indicating a general disagreement with those statements. 

There were notable differences between employees who had made one or more claims for psychological injuries and those who did not (13,013/14,868, 87.5%). Employees with a claims history were older (χ^2^(3) = 196.16, *p* < 0.001) and more often male (χ^2^(1) = 34.14, *p* < 0.001) than the remainder of the sample (Table 1). There were further differences in the sector distributions, χ^2^(3) = 100.99, *p* < 0.001, with a the majority of claimants being in the police force (1177/1855, 63.5%). About half of employees with a claims history reported being currently diagnosed with a mental health condition (941/1855, 50.7%) and most (1369/1855, 73.8%) reported having previously been diagnosed with a mental health condition. This is a notably greater proportion compared to the remainder of the sample, in which 18.5% (2412/13013) of employees reported being currently diagnosed with a mental health condition and 34.6% (4496/13013) reported having previously been diagnosed with a mental health condition. Similarly, while about a quarter of those who had not made a claim in the past reported having taken work-stress related leave (3593/13,013, 27.6%), over three quarters of those who indicated having made a workers’ compensation claim had done so (1410/1855, 76%). 

Table 1 further notes significant differences between subsamples on all stigma variables (except the shame dimension of the self-stigma scale), with Cohen’s *d* effect sizes in the small and small-to-medium ranges. For example, employees who underwent a claims process in the past reported having self-stigma burden (*M* = 0.24, *SD* = 1.08), whereas other employees reported neutral levels (*M* = 0.01, *SD* = 1.08), *F*(1) = 50.78, *p* < 0.001, *d* = 0.21. Additionally, employees with a claims history tended to disagree with the organisational commitment dimension (*M* = −0.61, *SD* = 0.80). In contrast, other employees agreed, on average, with the statements pertaining to organisational commitment (*M* = 0.24, *SD* = 0.73), *F*(1) = 278.80, *p* < 0.001, *d* = 0.41. While employees who had made claims disagreed with self-stigma items describing negative experiences with others (*M* = −0.25, *SD* = 1.12), they did so to a lesser extent than those who had not made claims (*M* = −0.58, *SD* = 1.07), *F*(1) = 106.54, *p* < 0.001, *d* = 0.31. Employees with a claims history had slightly more favourable views than other employees with regard to burden (*F*(1) = 12.58, *p* < 0.001, *d* = 0.09) and institutional stigma beliefs (*F*(1) = 10.74, *p* = 0.001, *d* = 0.08). In particular, claimants disagreed more strongly with items describing personal stigma beliefs of perceived burden (*M* = −0.88, *SD* = 0.88) and structural stigma at the workplace (*M* = −1.46, *SD* = 0.91) compared with non-claimants (*M* = −0.81, *SD* = 0.84 for perceived burden and *M* = −1.39, *SD* = 0.88 for structural stigma). 

Table 2 provides additional information comparing employees who indicated making a claim for work-related mental health problems with another subset of employees who indicated having had a diagnosed mental health condition but did not make a claim (4496/13,013, 34.6%). Around half of employees in both subgroups indicated experiencing a mental health condition at the time of survey completion (2412/4496, 53.6% and 941/1855, 50.7%, χ^2^(1) = 4.49, *p* = 0.034), and about half of those who indicated having been diagnosed with a mental health condition had taken stress leave (2132/4496, 47.4%) compared to three quarters of employees who had filed a claim (1410/1855, 76%, χ^2^(1) = 435.18, *p* < 0.001). Employees who had made a claim for psychological injuries obtained at work reported higher levels of self-stigma, personal stigma and perceived stigma at the workplace (*ps* < 0.01, *ds* ranged between 0.09 and 0.23), but reported lower levels of organisational commitment beliefs (*p* < 0.001, *d* = 0.26) compared to employees with a mental health condition who had not made a claim. 

### 3.2. Multiple Regression Models

The first model examined the influence of self-stigma, personal stigma, and workplace stigma dimensions on participants’ perceived stressfulness of their compensation claims process. The prediction model was significant, *F*(8, 1407) = 36.96, *p* < 0.001. As shown in Table 3, the combined predictors accounted for 17% of the variance of reported stress (*R*^2^ = 0.17, Adjusted *R*^2^ = 0.17). Mental health self-stigma with regard to experiences with others, perceived workplace stigma, and organisational commitment were significant unique predictors of participants’ stress experienced. Given the other variables in this model, organisational commitment had the largest unique contribution to stress experiences. Specifically, Beta weights indicated that for every 1 standard deviation increase in organisational commitment, stress experiences decreased by 0.3 of a standard deviation. For each 1 standard deviation increase in self-stigma expectations from others and perceived workplace stigma, stress experiences increased by 0.1 standard deviation. The proportion of total stress variance explained by organisational commitment was 12%. Perceived workplace stigma and mental health self-stigma with regard to experiences with others each contributed 8% to the total variance explained in Model 1. 

After the inclusion of potential confounding variables, the model explained 23% of the variance of reported stress (*R*^2^ = 0.23, Adjusted *R*^2^ = 0.22). While age group, gender, work sector, current mental health diagnosis, and stress leave significantly predicted stress, organisational commitment (*b* = −0.40, *SE-b* = 0.05, *β* = −0.25, *p* < 0.001, *r* = −0.36, *sr* = −0.21, *r_s_*^2^ = 0.56), perceived workplace stigma (*b* = 0.20, *SE-b* = 0.05, *β* = 0.12, *p* < 0.001, *r* = 0.29, *sr* = 0.10, *r_s_*^2^ = 0.37), and experiences with others (*b* = 0.13, *SE-b* = 0.04, *β* = 0.11, *p* = 0.001, *r* = 0.28, *sr* = 0.08, *r_s_*^2^ = 0.35) remained significant predictors of perceived stressfulness of the compensation claims process. 

Reported impact on participants’ recovery was also significantly predicted by stigma variables, *F*(8, 1407) = 38.21, *p* < 0.001. The second predictor model accounted for 18% of the variance of impact experiences (*R*^2^ = 0.18, Adjusted *R*^2^ = 0.17, see Table 4). Comparable to Model 1, organisational commitment contributed to more positive impact experiences in Model 2. For each 1 standard deviation increase in organisational commitment beliefs, impact scores leaned positive by 0.3 of a standard deviation. Self-stigma experiences with others and perceived workplace stigma, in contrast, contributed to negative impact experiences. With each standard deviation increase in these stigma scores, negative impact experiences increased by 0.1 standard deviation. In addition, the ignorance dimension of personal stigma experiences predicted a reduction in negative impact responses. This was contrary to the expected directionality in that a 1 standard deviation increase in personal stigma experiences was associated with a 0.1 standard deviation decrease in negative impact responses. Relative to the explained variance of impact scores, the contribution of organisational commitment was 14%. Perceived workplace stigma and mental health self-stigma with regard to experiences with others each contributed 7% to the explained variance in Model 2 and the ignorance dimension contributed 1%. 

With the addition of potential confounders, Model 2 accounted for 21% of the variance of impact experiences (*R*^2^ = 0.21, Adjusted *R*^2^ = 0.20). Age group, gender, and stress leave significantly predicted impact experiences. While the predictive effects of organisational commitment (*b* = −0.47, *SE-b* = 0.04, *β* = −0.30, *p* < 0.001, *r* = −0.39, *sr* = −0.25, *r_s_*^2^ = 0.71), experiences with others (*b* = 0.10, *SE-b* = 0.04, *β* = 0.09, *p* = 0.007, *r* = 0.27, *sr* = 0.06, *r_s_*^2^ = 0.34) and perceived workplace stigma (*b* = 0.12, *SE-b* = 0.05, *β* = 0.08, *p* = 0.010, *r* = 0.27, *sr* = 0.06, *r_s_*^2^ = 0.34) remained significant, ignorance (*b* = −0.08, *SE-b* = 0.06, *β* = −0.04, *p* = 0.192, *r* = −0.08, *sr* = −0.03, *r_s_*^2^ = 0.03) was not significant after the inclusion of additional covariates. 

The two stigma dimensions of self-stigma experiences with others and organisational commitment were further predictive of perceived fairness (Model 3, see Table 5) and support (Model 4, see Table 6) in the claims process. Both prediction models were significant (*F*(8, 1407) = 43.17, *p* < 0.001 and *F*(8, 1407) = 38.48, *p* < 0.001, respectively). The combined predictors accounted for 20% of the total variance of perceived fairness (*R*^2^ = 0.20, Adjusted *R*^2^ = 0.19) and 18% of the total variance of perceived support (*R*^2^ = 0.18, Adjusted *R*^2^ = 0.18). Organisational commitment beliefs had positive effects in Models 3 and 4, whereby a 1 standard deviation increase in organisational commitment accounted for a 0.4 standard deviation increase in perceived fairness and support. Self-stigma beliefs around experiences with others had small unique negative impacts on fairness and support, whereby a 1 standard deviation increase in experiences with others accounted for a 0.1 standard deviation decrease in fairness and support responses. The proportion of total variance explained by organisational commitment was 19% in Model 3 and 17% in Model 4. Mental health self-stigma with regard to experiences with others explained 6% of the total variance explained in Models 3 and 4. 

After including potential confounding variables, Model 3 accounted for 22% of the total variance of perceived fairness (*R*^2^ = 0.22, Adjusted *R*^2^ = 0.21) and Model 4 accounted for 19% of the total variance of perceived support (*R*^2^ = 0.19, Adjusted *R*^2^ = 0.18). In Model 3, age group, gender, and stress leave were significant predictors of perceived fairness, however, organisational commitment (*b* = 0.36, *SE-b* = 0.03, *β* = 0.39, *p* < 0.001, *r* = 0.44, *sr* = 0.33, *r_s_*^2^ = 0.88) and experiences with others (*b* = −0.07, *SE-b* = 0.02, *β* = −0.10, *p* = 0.002, *r* = −0.26, *sr* = −0.07, *r_s_*^2^ = 0.30) remained significant predictors of perceived fairness. Similarly, in Model 4, while gender and stress leave were significantly associated with perceived support, organisational commitment (*b* = 0.52, *SE-b* = 0.04, *β* = 0.37, *p* < 0.001, *r* = 0.42, *sr* = 0.32, *r_s_^2^* = 0.90) and experiences with others (*b* = −0.08, *SE-b* = 0.03, *β* = −0.08, *p* = 0.026, *r* = −0.24, *sr* = −0.05, *r_s_*^2^ = 0.29) remained significant predictors of perceived support. 

## 4. Discussion

In this article, we examined existing large-scale survey data of police and emergency services personnel to explore the impact of pursuing compensation claims because of psychological trauma, stress or a mental health condition sustained during the course of work. Firstly, we assessed claimants’ demographic characteristics in comparison with the remainder of the surveyed employees, and in comparison with a subset of employees who were diagnosed with a mental health condition in the past but did not report making a claim. Claimants were more often male, aged 45 years or over, and employed in the police sector than the other examined subsamples. Over two thirds of claimants reported having been formally diagnosed with a mental health condition and reported having taken sick leave due to stress more often than the other subsamples inspected. Half of employees who had made a claim for psychological injuries at work reported still having a mental health condition. This proportion was only slightly lower compared to those with a diagnosed mental health condition who had not filed a claim. Under the assumption that the compensation or claims process supports individuals with their recovery needs, it would have been expected that the gap between stated recovery rates would be greater. Indeed, there is some indication that the claims process places additional strain on employees, which affected their wellbeing and their sense of support. Employees with claims experience agreed with stigmatising beliefs to a greater extent, while also reporting a reduced sense of organisational commitment compared to employees who had been diagnosed with a mental health condition but had not filed a work claim. 

The presence of mental health stigma was associated with less favourable perceptions of the claims process, albeit to a small extent. When self-stigma, personal and workplace stigma dimensions were observed together, stigma accounted for around 20% of the variance in claims experiences and its impact on recovery. In comparison, previous research conducted with American individuals who were in psychiatric treatment found that two self-stigma facets predicted 26% of the variance in participants’ personal recovery scores [28]. It is therefore possible that the influence of stigma on recovery is greater than on the claims experience. This should be further examined in purpose-designed investigations. 

The self-stigma dimension of experiences with others was uniquely associated with heightened negative impact and reduced feelings of support through the claims process. It is possible that this aspect of stigma was influential because, for the majority of claimants, it reflected lived experiences of discrimination (i.e., “people have treated me unfairly because of my mental health problem”) rather than prejudiced beliefs (i.e., “people would treat me unfairly because of my mental health problem”). This suggest that the relevance of stigma may be particularly pronounced when people witness behavioural components of stigma such as anger or avoidance from others. This is consistent with previous research indicating that experience of discrimination hindered social and vocational integration among individuals with depression [29]. A practical implication of this finding may be that claimants who experience negative interactions due to their mental health condition may benefit from engaging a mediator who can navigate potentially upsetting negotiations with employers and managers on the claimant’s behalf [30]. 

The workplace stigma dimension of perceived organisational commitment was associated with claims experiences across all considered outcomes. Higher agreement with items on this dimension likely reflected positive experiences with superiors and colleagues, and positive attitudes pertaining to a supportive organisational culture. Importantly, the protective effects of organisational commitment beliefs were of greater magnitude than the negative effects of stigmatising mental health beliefs, making the provision of high-quality institutional support a possible focal point of future organisational-level interventions aimed at strengthening the compensation system. To improve employee beliefs of organisational commitment, it may be necessary to address several key aspects of workplace culture, including level of supervisor support, management style, workplace communication and openness to discussing emotional issues [31,32]. Furthermore, it is possible that greater visibility of return-to-work processes resulting in the continuation of productive career paths would aid in de-stigmatisation efforts through modelling successful reintegration. This could be tested by extending existing manager training to facilitate return-to-work after illness or injury [33] to take an institution-wide approach to involve all employees. Similar methods have yielded promising results, for example, Stelling and colleagues found that voluntary faculty-level disclosures of resident physicians’ mental health problems had destigmatising effects on junior physicians [34]. A recent review article of Australian initiatives aimed at reducing stigma, however, suggests that institutional- and structural-level interventions are still lacking and no firm conclusions about their effectiveness can be drawn to date [35]. Further research is therefore advised. 

Further stigma dimensions uniquely influenced the reported impact that the workers’ compensation claims process had on their recovery from mental illness or psychological injury. There was a small but significant negative impact of perceived workplace stigma from other employees on the recovery process, which suggested that the perceived attitudes from co-workers and managers may extend to experiences of workers’ recovery process. Ignorance about mental health was further related to recovery, whereby ignorance unexpectedly exerted a positive influence. However, the latter finding was not robust to inclusions of demographic and mental health history variables, suggesting that ignorance about mental health shared common variance with non-stigma variables that explained the detected associations with recovery experiences [33]. Consequently, out of the eight mental health stigma dimensions considered, only the dimensions of organisational commitment, experiences with others, and perceived workplace stigma showed significant unique associations with compensation claims experiences and recovery after accounting for claimants’ demographic characteristics and mental health history. 

Strengths and limitations of this research should be noted. A key strength of the current research is that personal, workplace, and self-dimensions of stigma were assessed together to examine the combined and unique effects of stigma types on workers’ claims process and recovery. By doing so, an assessment of the overall influence of stigma as well as the main unique contributors to workers’ experiences was possible. To our knowledge, this is the first examination of this kind. In addition, the current research utilises large-scale survey data of Australian police and emergency service personnel. It can therefore be reasonably assumed that the responses and demographic characteristics presented in this article are generalisable to the wider population in question. 

Limitations of this research are the exploratory and cross-sectional nature of analyses, scale constructions, and the date of the original assessment. This research was conducted on archival data originally collected in 2017 and 2018. The survey was not designed to test the specific research aims outlined in this work. Consequently, we selected measures a posteriori, which were not optimal to test the proposed relations investigated in this study. To illustrate, temporal sequencing of independent and dependent variables in a longitudinal design would have allowed for causal inferences to be made. Additionally, the outcome measures assessing participants experiences during their claims process were all single-item indicators that captured the underlying global constructs of stress, impact, support and recovery but may not have captured all facets of interest. For example, the assessment of various kinds of stress such as tension, irritability, or inability to unwind would allow for a more nuanced observation of the consequences of stigma on claimants’ experiences. Relatedly, the items utilised in this research were not established measures and not pre-tested for their psychometric properties, limiting the ability to gauge construct validity [36]. Lastly, data collection took place before the Black Summer bush fire season in 2019 and 2020 and before the global coronavirus pandemic, both of which severely impacted the police and emergency service sectors. Therefore, it is possible that various changes in the work sectors and cohorts have taken place that are not captured in this article.

## 5. Conclusions

The current examination suggested that mental health stigma explained about one fifth of the variances in the claims and compensation process experiences for work-related psychological injuries of police and emergency service personnel. Among the stigma dimensions assessed, the self-stigma dimension of experiences with others and perceived organisational commitment were the most influential. These results suggest that multi-level interventions that go beyond interventions targeted at the individual who experienced a work-related psychological injury to include colleagues and managers may prove particularly useful in alleviating negative effects attributable to mental health stigma. Given the pivotal role first responders play in our communities and in society at large, this article highlighted the potential of shifting institutional parameters to help the first responder workforce navigate the distressing landscape of psychological injury.

## Figures and Tables

**Table 1:** Sample Characteristics and Between-Group Statistics on Key Variables, Comparing Employees who reported Making One or More Claims with Employees who have never made a Claim for Psychological Injuries Obtained at Work.

	Employees Who Did Not Indicate Previous Claim (*N* = 13,013)		Employees with Claim History (1 or More) (*N* = 1855)				
Variable	*M/n*	*SD/%*	*M/n*	*SD/%*	*F/*χ^2^	*p*	*w/g*
Age group (years, *n*, %)					196.16	<0.001	0.81
<35	2883	22.2	176	9.5	-	-	-
35–44	3590	27.6	475	25.6	-	-	-
45–54	4369	33.6	795	42.9	-	-	-
≥55	2171	16.7	409	22.0	-	-	-
Gender (*n*, %)					34.14	<0.001	0.58
Female	5294	40.7	623	33.6	-	-	-
Male	7719	59.3	1232	66.4	-	-	-
Sector (*n*, %)					100.99	<0.001	0.58
Ambulance	3050	23.4	423	22.8	-	-	-
Fire and Rescue	2745	21.1	230	12.4	-	-	-
Police	6911	53.1	1177	63.5	-	-	-
State Emergency Services	307	2.4	25	1.3	-	-	-
Mental Health History (*n*, %)							
Previous diagnosis	4496	34.6	1369	73.8	338.96	<0.001	1.84
Condition currently present	2412	18.5	941	50.7	103.80	<0.001	0.72
Ever taken work stress leave	3593	27.6	1410	76.0	1703.48	<0.001	4.13
Self-stigma ^◌^							
Shame	0.27	1.05	0.29	1.07	0.32	0.571	0.02
Burden	0.01	1.08	0.24	1.08	50.78	<0.001	0.21
Experiences with others	−0.58	1.07	−0.25	1.12	106.54	<0.001	0.31
Personal Stigma							
Ignorance	−1.26	0.67	−1.36	0.67	32.30	<0.001	0.14
Perceived burden	−0.81	0.84	−0.88	0.88	12.58	<0.001	0.09
Workplace Stigma							
Perceived stigma	0.25	0.79	0.54	0.82	223.23	<0.001	0.37
Structural stigma	−1.39	0.88	−1.46	0.91	10.74	0.001	0.08
Organisational commitment	0.24	0.73	−0.06	0.80	278.80	<0.001	0.41

Note. **^◌^** self-stigma items were only presented if participants indicated to have a diagnosed or undiagnosed mental health condition; *w* = Cohen’s *w* effect size; *g* = Hedges *g* effect size.

**Table 2:** Sample Characteristics and Between-Group Statistics on Key Variables, Comparing Employees who reported Making One or More Claims with Employees who have Had a Diagnosed Mental Health Condition but Did not Make a Claim.

	Employees with a Mental Health Condition (*N* = 4496)		Employees with Claim History (1 or More) (*N* = 1855)				
Variable	*M/n*	*SD/%*	*M/n*	*SD/%*	*F*/χ^2^	*p*	*w/g*
Age group (years, *n*, %)					140.40	<0.001	0.68
<35	894	19.9	176	9.5	-	-	-
35–44	1308	29.1	475	25.6	-	-	-
45–54	1592	35.4	795	42.9	-	-	-
≥55	702	15.6	409	22.0	-	-	-
Gender (*n*, %)					121.40	<0.001	1.10
Female	2189	48.7	623	33.6	-	-	-
Male	2307	51.3	1232	66.4	-	-	-
Sector (*n*, %)					99.42	<0.001	0.58
Ambulance	1073	23.9	423	22.8	-	-	-
Fire and Rescue	958	21.3	230	12.4	-	-	-
Police	2338	52.0	1177	63.5	-	-	-
State Emergency Services	127	2.8	25	1.3	-	-	-
Mental Health History (*n*, %)							
Previous diagnosis	4496	100	1369	73.8	527.54	<0.001	2.30
Condition currently present	2412	53.6	941	50.7	97.58	<0.001	0.99
Ever taken work stress leave	2132	47.4	1410	76.0	435.18	<0.001	2.09
Self-stigma ^◌^							
Shame	0.19	1.05	0.29	1.07	9.19	0.002	0.09
Burden	0.04	1.08	0.24	1.08	40.12	<0.001	0.19
Experiences with others	−0.51	1.07	−0.25	1.12	64.85	<0.001	0.25
Personal Stigma							
Ignorance	−1.43	0.62	−1.36	0.67	15.20	<0.001	0.11
Perceived burden	−1.01	0.82	−0.88	0.88	29.88	<0.001	0.15
Workplace Stigma							
Perceived stigma	0.36	0.80	0.54	0.82	68.33	<0.001	0.23
Structural stigma	−1.46	0.88	−1.46	0.91	0.02	0.881	0.00
Organisational commitment	0.14	0.75	−0.06	0.80	88.18	<0.001	0.26

Note. **^◌^** self-stigma items were only presented if participants indicated to have a diagnosed or undiagnosed mental health condition; *w* = Cohen’s *w* effect size; *g* = Hedges *g* effect size.

**Table 3:** Standard Multiple Regression Results for Self-stigma, Personal Stigma, and Workplace Stigma Predicting Police and Emergency Service Personnel’s Perceived Stress ^a^ Going Through the Claims Process for Psychological Injuries.

Model	*b*	*SE-b*	Beta	Pearson *r*	*sr*	*r_s_* ^2^	*p*
Constant	3.07	0.09					
Self-stigma—shame	−0.01	0.04	−0.01	0.16	−0.01	0.15	0.802
Self-stigma—burden	0.08	0.04	0.06	0.23	0.05	0.30	0.063
Self-stigma—experiences with others **	0.12	0.04	0.10	00.28	0.08	0.45	0.002
Personal Stigma—ignorance	−0.10	0.06	−0.05	−0.08	−0.04	0.03	0.130
Personal Stigma—perceived burden	−0.08	0.04	−0.06	−0.04	−0.05	0.01	0.065
Workplace Stigma—perceived stigma ***	0.20	0.05	0.13	0.29	0.12	0.48	<0.001
Workplace Stigma—structural stigma	−0.05	0.04	−0.03	−0.09	−0.04	0.04	0.181
Organisational Commitment ***	−0.40	0.05	−0.25	−0.36	−0.23	0.73	<0.001

Note. ^a^ Higher values on the stress measure indicate worse outcomes for participants. *R*^2^ = 0.17, Adjusted *R*^2^ = 0.17. *sr* is the semi-partial correlation. *r_s_*^2^ is the squared structure coefficient. ** *p* < 0.01, *** *p* < 0.001, *N* = 1416.

**Table 4:** Standard Multiple Regression Results for Self-stigma, Personal Stigma, and Workplace Stigma Predicting Police and Emergency Service Personnel’s Reported Impact ^a^ of the Claims Process for Psychological Injuries on their Recovery.

Model	*b*	*SE-b*	Beta	Pearson *r*	*sr*	*r_s_* ^2^	*p*
Constant	3.41	0.09					
Self-stigma—shame	0.02	0.04	0.02	0.17	0.02	0.17	0.523
Self-stigma—burden	0.05	0.04	0.04	0.22	0.03	0.26	0.226
Self-stigma—experiences with others **	0.10	0.04	0.09	0.26	0.07	0.39	0.008
Personal Stigma—ignorance *	−0.14	0.06	−0.07	−0.08	−0.06	0.03	0.022
Personal Stigma—perceived burden	−0.03	0.04	−0.02	−0.01	−0.02	0.01	0.529
Workplace Stigma—perceived stigma **	0.13	0.05	0.08	0.27	0.08	0.40	0.005
Workplace Stigma—structural stigma	−0.04	0.04	−0.02	−0.08	−0.03	0.03	0.333
Organisational Commitment ***	−0.47	0.04	−0.30	−0.39	−0.27	0.83	<0.001

Note. ^a^ Higher scores on the recovery impact measure indicate greater negative impact. *R*^2^ = 0.18, Adjusted *R*^2^ = 0.17. *sr* is the semi-partial correlation. *r_s_*^2^ is the squared structure coefficient. * *p* < 0.05, ** *p* < 0.01, *** *p* < 0.001, *N* = 1416.

**Table 5:** Standard Multiple Regression Results for Self-stigma, Personal Stigma, and Workplace Stigma Predicting Police and Emergency Service Personnel’s Reported Fair Treatment ^a^ Going Through the Claims Process for Psychological Injuries.

Model	*b*	*SE-b*	Beta	Pearson *r*	*sr*	*r_s_* ^2^	*p*
Constant	1.94	0.05					
Self-stigma—shame	0.01	0.02	0.02	−0.14	0.01	0.10	0.619
Self-stigma—burden	0.01	0.02	0.01	−0.17	0.01	0.14	0.673
Self-stigma—experiences with others **	−0.07	0.02	−0.10	−0.25	−0.08	0.32	0.002
Personal Stigma—ignorance	0.04	0.04	0.03	0.02	0.03	0.00	0.294
Personal Stigma—perceived burden	−0.01	0.03	−0.01	−0.04	−0.01	0.01	0.816
Workplace Stigma—perceived stigma	−0.04	0.03	−0.04	−0.25	−0.04	0.30	0.151
Workplace Stigma—structural stigma	−0.02	0.02	−0.02	0.03	−0.02	0.00	0.430
Organisational Commitment ***	0.36	0.03	0.39	0.43	0.35	0.95	<0.001

Note. ^a^ Higher values on the fair treatment measure indicate better outcomes for participants. *R*^2^ = 0.20, Adjusted *R*^2^ = 0.19. *sr* is the semi-partial correlation. *r_s_*^2^ is the squared structure coefficient. ** *p* < 0.01, *** *p* < 0.001, *N* = 1416.

**Table 6:** Standard Multiple Regression Results for Self-stigma, Personal Stigma, and Workplace Stigma Predicting How Supportive ^a^ Police and Emergency Service Personnel Found their Claims Process for Psychological Injuries.

Model	*b*	*SE-b*	Beta	Pearson *r*	*sr*	*r_s_* ^2^	*p*
Constant	2.20	0.08					
Self-stigma—shame	−0.00	0.03	−0.00	−0.14	−0.00	0.12	0.974
Self-stigma—burden	−0.00	0.03	−0.00	−0.17	−0.00	0.16	0.980
Self-stigma—experiences with others *	−0.08	0.03	−0.08	−0.24	−0.07	0.31	0.014
Personal Stigma—ignorance	0.08	0.05	0.04	0.02	0.04	0.00	0.166
Personal Stigma—perceived burden	−0.05	0.04	−0.04	−0.05	−0.03	0.01	0.206
Workplace Stigma—perceived stigma	−0.03	0.04	−0.02	−0.22	−0.02	0.28	0.413
Workplace Stigma—structural stigma	0.04	0.03	0.03	0.07	0.03	0.03	0.249
Organisational Commitment ***	0.51	0.04	0.36	0.41	0.33	0.94	<0.001

Note. ^a^ Higher values on the support measure indicate better outcomes for participants. *R*^2^ = 0.18, Adjusted *R*^2^ = 0.18. *sr* is the semi-partial correlation. *r_s_*^2^ is the squared structure coefficient. * *p* < 0.05, *** *p* < 0.001, *N* = 1416.

## Data Availability

The confidentialised Unit Record File for *Answering the Call* is available on application to Beyond Blue. To protect the privacy of individuals and organisations who participated in the study, access is restricted to researchers who have formal approval from the Institutional Review Board or equivalent of their institution and who agree to the terms and conditions as specified by Beyond Blue.
